# Diagnosis of Spontaneous Cerebrospinal Fluid Rhinorrhea Using Thin-Slice Computed Tomographic Images With Multiplanar Reconstruction and Three-Dimensional Virtual View Endoscopy

**DOI:** 10.7759/cureus.26868

**Published:** 2022-07-14

**Authors:** Tomoya Yamaguchi, Tatsunori Sakamoto, Toru Miwa, Hayato Tabu, Shin-ichi Kanemaru

**Affiliations:** 1 Otolaryngology - Head and Neck Surgery, Tazuke Kofukai Medical Research Institute, Kitano Hospital, Osaka, JPN; 2 Otolaryngology, Shimane University Faculty of Medicine, Shimane, JPN; 3 Otolaryngology - Head and Neck Surgery, Kyoto University, Kyoto, JPN; 4 Neurology, Tazuke Kofukai Medical Research Institute, Kitano Hospital, Osaka, JPN

**Keywords:** trans-nasal endoscopic surgery, virtual endoscopic view image, image reconstruction, thin-slice ct, spontaneous cerebrospinal fluid rhinorrhea

## Abstract

Spontaneous cerebrospinal fluid (CSF) rhinorrhea represents an important clinical entity, which is associated with elevated intracranial pressure and is rarely treated successfully without surgical intervention. Here we report a case of spontaneous CSF rhinorrhea. The patient was a 54-year-old male, who presented with bacterial meningitis and was referred to the Department of Otorhinolaryngology for a detailed examination of the nose and sinuses. Reconstructed thin-slice computed tomography (CT) revealed multiple fistulae on the clivus. The defect was successfully repaired by transnasal endoscopic surgery, with the assistance of virtual endoscopic images, which were created by the surgical planning and navigation system from thin-slice CT images. This incremental improvement in the imaging technique helped with the diagnosis and surgical treatment of CSF rhinorrhea.

## Introduction

Cerebrospinal fluid (CSF) rhinorrhea is a rare condition resulting from a fistula between the subarachnoid space and the paranasal sinus. The typical clinical appearance of CSF rhinorrhea includes unilateral rhinorrhea (90%), meningitis (6%), and a unilateral polyp (1%) [1]. CSF rhinorrhea is most commonly associated with head injuries, skull base tumors, and surgical trauma typically from sinus surgery. Other less common causes, such as hydrocephalus, empty sella syndrome, and spontaneous CSF rhinorrhea, have also been reported [2]. The prevalence of spontaneous CSF rhinorrhea varies between 4% and 40% among reports [2]. In cases with intracranial infection, it is critically necessary to identify and seal the fistula. However, it is not always easy to recognize the location of the leak preoperatively. In this report, we describe a case of spontaneous CSF rhinorrhea, in which the fistula was predicted by multiplanar reconstructed (MPR) images and three-dimensional (3D) virtual endoscopic view images created from the preoperative thin-slice computed tomography (CT).

## Case presentation

Case description

We present here the case of a 54-year-old man with bacterial meningitis. His chief complaints were headache, chills, and numbness in both hands. He has a past history of hypertension, and a history of endoscopic sinus surgery performed in another hospital four years ago. The patient currently has a two-week history of nasal discharge, sore throat, and frequent nose blowing before the consultation. Approximately 4 hours before visiting the hospital, the patient developed a headache with a gradual tightening sensation at the back of the head, with accompanying chills, arthralgia, and numbness in both hands.

At the time of consultation in the Emergency Room of our hospital, the patient was conscious, with a blood pressure of 180/140 mmHg. The heart rate was 94 beats/min. The SpO_2_ at room air was 96%, and the body temperature was 39.2 degrees Celsius. Initially, no significant neurological findings were observed, except that his consciousness deteriorated to E4V2M6 (Glasgow Coma Scale 12) during the examination.

The labs revealed that his C-reactive protein (CRP) level was 5.81 mg/dL and white blood cell count was 9100 cells/μL. CSF sampling indicated a normal glucose level (70 mg/dL), but the number of nucleated cells was elevated to 2039 cells/μL, and the protein level in the CSF was elevated to 2891.7 mg/dL. These findings strongly suggested the presence of bacterial meningitis.

**Figure 1:**
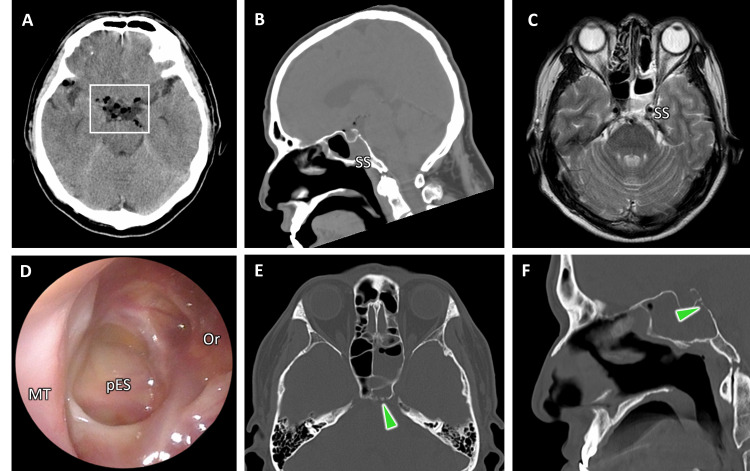
Findings at the consultation to the ENT (A) The head CT showed regions of air in the prepontine cistern (white box). (B) A partial soft tissue area was observed in the left SS. The skull base fistula was not identifiable. (C) The head MRI (T2WI) showed no mass lesion. A partial high-intensity area was observed in the left SS. (D) A pulsation and transparent viscous discharge was found in the pES. (E, F) Multiplanar reconstructed axial (E) and coronal images (F) of thin-slice CTs showed multiple bone defects (arrowheads). SS: sphenoid sinus; MT: middle turbinate or medial orbital wall; pES: posterior ethmoid sinus; T2WI: T2-weighted imaging.

The 4-mm slice width head CT images revealed pneumocephalus with air accumulation in the right Sylvian fissure, suprasellar cistern, and left ambient cistern (Figure 1A). An area of soft tissue density was observed in the left sphenoid sinus. No obvious communication between the nasal and the cranial cavities was identified on the CT (Figure 1B). Magnetic resonance imaging showed no mass lesions, which can cause a fistula (Figure 1C).

CSF rhinorrhea was suspected from the patient’s history of sinus surgery and the nasal symptoms before the consultation, and he was referred to our department. A fiberscopic examination of his left nasal cavity revealed a yellowish crust and pulsation in the postoperative ethmoidal cavity (Figure 1D). It was difficult to inspect the ostium of the sphenoid. The results of the bacterial culture from the left nasal cavity showed no significant bacterial growth. His level of consciousness and headache improved gradually after three days of treatment with meropenem (MEPN) and linezolid (LZD). However, the watery rhinorrhea continued. On the third day after admission, the CRP level increased to 15.04 mg/dL. A thin-slice CT of the sinuses (slice thickness was 0.5 mm) was taken, and the MPR images revealed multiple bone defects on the clivus (Figure 1E, 1F). Thus, an endoscopic repair of the CSF rhinorrhea was planned.

Intraoperative findings

**Figure 2:**
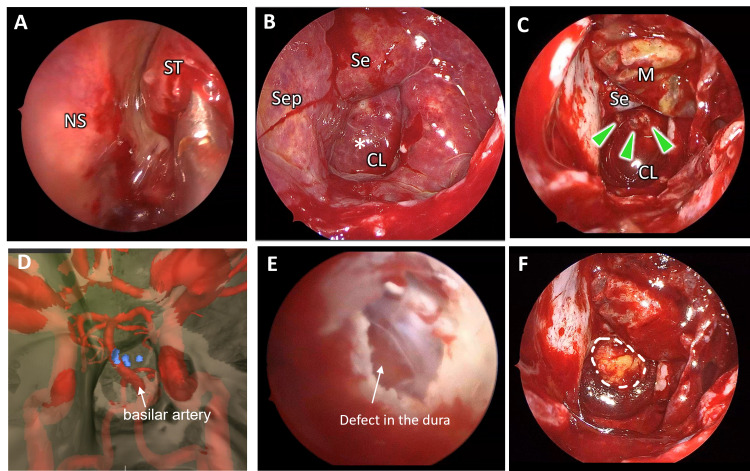
Intraoperative endoscopic images and the virtual endoscopic view (A) The outflow of a colorless and transparent serum from the sphenoid ostium was seen. (B) The fistula was seen on the posterior medial side of the clivus (CL) (asterisk). (C) Multiple bone defects were found on the CL (arrowheads). (D) The virtual endoscopic view image created from the thin-slice CT and MRI successfully visualized the multiple bone defects (blue dots) and the basilar artery in the cranium. (E) The defect in the dura was seen from the bone window. (F) Abdominal fat was grafted using the bath-plug method (white dots lines). NS: nasal septum; ST: superior turbinate; Se: sella turcica; Sep: sphenoid septum; M: sphenoid mucosal flap; CL: clivus.

First, we inspected the previous surgical sites in the anterior and posterior ethmoidal cavities and the sphenoid ostium. A pulsatile outflow of a transparent fluid was observed from the pin-hole sphenoid ostium (Figure 2A). The superior turbinate was resected and the sphenoid ostium was enlarged. The sphenoid sinus was filled with a transparent liquid, and the mucosa was diffusely thickened. A fine, 1-mm-wide fistula with pulsatile leakage of CSF was observed on the medial side of the clivus (Figure 2B).

After resection of the lower half of the middle turbinate and a wide sphenoidotomy, the mucosa of the sphenoid septum and the clivus were moved laterally as a sphenoid mucosal flap (Figure 2C), and multiple bony defects were exposed. These defects corresponded to the bone defects visualized on the 3D virtual endoscopic view images, which were created by the surgical planning and navigation system (Stealth Station ENT®, Medtronic, Dublin, Ireland) and it was very helpful in predicting the location of bone defects (Figure 2D). The system also visualized and alerted us to the basilar artery running inside the cranial cavity close to the fistula (Figure 2D). A small split of the dura mater was observed through the bone window (Figure 2E). The bony defects were carefully trimmed to make a single hole, abdominal fat was inserted into the defect using the bath-plug method [3], and then fixed with fibrin glue (Figure 2F). The water-tight closure was confirmed by increasing the intrathoracic pressure (the Valsalva method). The clivus was further covered with additional abdominal fat and the sphenoid mucosal flap.

Postoperative course

The initial antibiotic regimen, MEPN and LZD, was revised to MEPN after the surgery, further changed to ampicillin sulbactam on day 15, and continued until day 22, according to the result of a bacterial culture and a sensitivity test. The patient’s headache improved within seven days postoperatively. The lab test on day 14 was negative for inflammatory reactions. The postsurgical course was uneventful, and the patient was discharged on day 27. During the 13 months of follow-up, he was free from symptom recurrence such as transparent rhinorrhea, fever, and headache.

## Discussion

Initially, the cause of the CSF rhinorrhea in this case was presumed to be surgical damage from the endoscopic sinus surgery four years prior. However, intraoperative findings and preoperative images revealed that the fistulae could not be reached with the instruments normally used for nasal surgery. The anterior wall and the ostium of the sphenoid sinus were intact during our surgery. Also, in the image analyses of the preoperative thin-slice CT, the straight line through the bottom of the sphenoid ostium and the bottom of the sella reached the clivus below the fistulae. This indicated the need for a curved instrument to reach the location. Moreover, the length of this line from the clivus to the inner surface of the nasal bone was 7.49 cm. The length of the straight part of the commonly used nasal forceps is 8 cm or more, which could not possibly be inserted within this space. Thus, we concluded that the current CSF rhinorrhea was spontaneous, instead of iatrogenic. Notably, the MPR images via thin-slice CT preoperatively were useful for this diagnosis of CSF rhinorrhea.

In this case, we identified “clivus” as a precise location of CSF rhinorrhea via 3D endoscopic view images intraoperatively. Spontaneous CSF rhinorrheas are most commonly found in the roof of the ethmoid, the lamina cribrosa, and the lateral wall of the sphenoid sinus, and less commonly in the clivus (9%) [4]. As a material for reconstruction of the defect, single or multilayered reconstruction, pliable or rigid methods, free mucosal grafts, synthetic grafts, fascial or fat grafts, and vascularized tissue were considered. Although vascularized flap such as nasoseptal flap (NSF) has been proven to be superior in widespread defects due to skull base tumor surgery, etc., small (<1 cm^2^) defects are reliably closed in over 90% of cases using multilayered free grafts, obviating the need for vascularized flap reconstruction, and in patients with spontaneous CSF leaks, there is no consensus within the literature regarding the best material. There was also the option of using a vascularized flap like NSF, but in this case, the defect was small and the bath-plug seemed to be suitable which was easy to adjust the size [5]. We used a bath-plug and confirmed the closure with the Valsalva method during the operation [6]. We used a sphenoid mucosa flap for covering the bath-plug. Only sphenoid sinus mucosa was not enough to provide adequate blood flow in the graft, but we expected that epithelialization would be accelerated. In fact, a fiberscopic examination showed that epithelialization inside the sphenoid sinus was done completely three months after surgery [6]. Three years have passed without recurrence since the surgery. However, it is reported that 18% of patients required revision surgery with a mean follow-up time of greater than 10 years, and we need more follow-up.

## Conclusions

We experienced a case of spontaneous CSF rhinorrhea, for which the point of leakage was identified on the reconstructed preoperative thin-slice CT. The defect was successfully repaired by transnasal endoscopic surgery, with the assistance of virtual endoscopic images, which were created by the surgical planning and navigation system from thin-slice CT images. In the present case, it was not possible to identify the skull base fistula on the 4-mm slice width head CT or MRI. However, the MPR and the 3D virtual endoscopic view images using the 0.5-mm thin-slice CT in combination with MRI visualized the fistula as well as the basilar artery in the cranium. In addition, postoperative analysis of the length to clivus on the thin-slice CT revealed the cause of CSF rhinorrhea. Our case indicates the importance of using imaging technology to facilitate the diagnosis of skull base fistulae. This incremental improvement in the imaging technique helped with the diagnosis and surgical treatment of CSF rhinorrhea.
